# Validation of a modified version of the gross motor function measure in *PPPR5D* related neurodevelopmental disorder

**DOI:** 10.1186/s13023-024-03067-3

**Published:** 2024-02-07

**Authors:** Cara H. Kanner, David Uher, Kyle Zreibe, Gabriella Beard, Madison Patterson, Matthew Harris, Jerome Doerger, Sean Calamia, Wendy K. Chung, Jacqueline Montes

**Affiliations:** 1 Department of Rehabilitation and Regenerative Medicine, Columbia University Irving Medical Center, 617 West 168th Street, New York, NY 10032 USA; 2 Department of Biobehavioral Sciences, Teachers College, Columbia University, New York, NY USA; 3 Department of Rehabilitation, UHealth-Jackson Holtz Children’s Hospital, Miami, FL USA; 4 Department of Pediatrics, Columbia University Irving Medical Center, New York, NY USA; 5 Department of Pediatrics, Boston Children’s Hospital and Harvard Medical School, Boston, MA USA

**Keywords:** Outcome measures, Clinical trials, Motor function, Neurodevelopmental disorders, Natural history, Gross motor function measure (GMFM), *PPP2R5D*

## Abstract

**Background:**

Protein phosphatase 2 regulatory subunit B’ Delta (PPP2R5D)-related neurodevelopmental disorder is a rare genetic condition caused by pathogenic variants in the *PPP2R5D* gene. Clinical signs include hypotonia, gross motor delay, intellectual disability (ID), epilepsy, speech delays, and abnormal gait among other impairments. As this disorder was recognized within the last decade, there are only 103 people published diagnoses to date. A thorough understanding of the motor manifestations of this disorder has not yet been established. Knowledge of the natural history of *PPP2R5D* related neurodevelopmental disorder will lead to improved standard of care treatments as well as serve as a baseline foundation for future clinical trials. Appropriate outcome measures are necessary for use in clinical trials to uniformly measure function and monitor potential for change. The aim of this study was to validate the gross motor function measure (GMFM) in children and adults with *PPP2R5D*-related neurodevelopmental disorder in order to better characterize the disorder.

**Results:**

Thirty-eight individuals with *PPP2R5D* pathogenic variants, median age 8.0 years (range 1–27) were evaluated. Gross motor, upper limb and ambulatory function were assessed using the GMFM-66, six-minute walk test (6MWT), 10-meter walk run (10MWR), timed up and go (TUG), and revised upper limb module (RULM). The pediatric disability inventory computer adapted test (PEDI-CAT) captured caregiver reported assessment. Median GMFM-66 score was 60.6 (SD = 17.3, range 21.1–96.0). There were strong associations between the GMFM-66 and related mobility measures, 10MWR (r_s_ = −0.733; *p* < 0.001), TUG (r_s_= −0.747; *p* = 0.003), 6MWT (r = 0.633; *p* = 0.006), RULM (*r* = 0.763; *p* < 0.001), PEDICAT-mobility (r = 0.855; *p* < 0.001), and daily activities (r = 0.822; *p* < 0.001) domains.

**Conclusions:**

The GMFM is a valid measure for characterizing motor function in individuals with *PPP2R5D* related neurodevelopmental disorder. The GMFM-66 had strong associations with the RULM and timed function tests which characterized gross motor, upper limb and ambulatory function demonstrating concurrent validity. The GMFM-66 was also able to differentiate between functional levels in *PPP2R5D* related neurodevelopmental disorder demonstrating discriminant validity. Future studies should examine its sensitivity to change over time, ability to identify sub-phenotypes, and suitability as an outcome measure in future clinical trials in individuals with *PPP2R5D* variants.

**Supplementary Information:**

The online version contains supplementary material available at 10.1186/s13023-024-03067-3.

## Background

Protein phosphatase two regulatory subunit B’ delta (PPP2R5D)- related neurodevelopmental disorder is caused by pathogenic variants in the *PPP2R5D* gene which commonly results from *de novo* missense variants [1]. The *PPP2R5D* gene, located on chromosome 6, is part of the phosphatase-2A (PPP2A) family of phosphatases with critical roles in development. It also helps maintain neurons and regulates neuronal signaling [2]. Genetic variants of *PPP2R5D* were recognized in 2015 to be associated with intellectual disability (ID)(OMIM#616355) [3]. As of 2023, there were 103 people with *PPP2R5D* related neurodevelopmental disorder published, with 16 different genetic variants [4, 5]. Clinical manifestations commonly include hypotonia, gross motor delay, ID, and macrocephaly. Some individuals, (27.7%), with *PPP2R5D* variants have been co-diagnosed with autism spectrum disorder (ASD) [4, 6, 7]. The condition has also been associated with Parkinsonism [8]. There is a spectrum of disease phenotype that is still being described.

Outcome measures will be needed to assess natural history and effects of new treatments in clinical trials for neurogenetic conditions [9]. Characterizing motor function with reliable outcome measures will be an important dimension of neurological function to include.

The gross motor function measure (GMFM) is an 88-item functional assessment for evaluating gross motor change over time, originally validated for cerebral palsy (CP) [10]. It was subsequently shown to be reliable and valid for use in children with Down syndrome (DS) and spinal muscular atrophy (SMA). Validation in these populations further establishes its use in a similar population with low tone and that may be either non-ambulatory or ambulatory [11, 12]. Other studies have also included adult participants with rare genetic and neuromuscular disorders measured using the GMFM [13, 14]. Although the GMFM has not yet been validated for use in individuals with this rare genetic disease, many of the impairments seen in individuals diagnosed with *PPP2R5D* related neurodevelopmental disorder closely resemble those with SMA and DS, specifically hypotonia and developmental delay. A modified version, the GMFM-66, was developed using Rasch analysis, for improved scoring and data interpretation and contains only 66 of the original 88 items. Items from each of the five dimensions; (A) lying and rolling, (B) sitting, (C) creeping and kneeling, (D) standing, (E) walking, running, and jumping are combined into a total score using the Gross Motor Ability Estimator (GMAE). Items that are incomplete on the GMFM-66 are able to be scored as missing rather than zero [15]. Additionally, several abbreviated approaches have been used to further reduce the items administered, to decrease administration burden and time. It was found that only 13 items are necessary to accurately estimate gross motor function [16]. The ‘Basal-Ceiling’ approach (GMFM B&C) is a similar method to that used in other norm-referenced tests where individuals begin at a specific start and end point based on their abilities [17]. The ‘Item Set’ approach (GMFM-66-IS) screens for function based on an initial decision item and then utilizes one of four items sets, ranging from 15 to 39 total items, which are better related to an individual’s current functional level [18].

The aims of this study were to (1) determine the concurrent and discriminant validity of the GMFM in children and adults with PPP2R5D related neurodevelopmental disorder, in order to (2) characterize motor function in this group of individuals.

## Method

### Participants

Individuals with confirmed pathogenic variants in *PPP2R5D* were invited to participate in research activities offered at a family meeting in July 2022 as part of a larger natural history study with various specialties collaborating to characterize rare genetic disorders with approval by Columbia University Irving Medical Center’s Institutional Review Board #AAAT8830.

Prior to the meeting, caregivers provided information regarding medical history, development, and current level of mobility. Caregiver reported ambulatory status was reported as either ambulatory, uses an assistive device, uses a wheelchair part-time, or uses a wheelchair full-time. At the meeting, individuals completed a comprehensive 90 minute motor function assessment which consisted of upper limb, gross motor, and ambulatory function. Participants were initially screened and grouped by Gross Motor Function Classification System (GMFCS), which describes five levels of motor function based on functional abilities and limitations ranging from level I (most able) to level V (most limited) [19]. GMFCS scores were used to split participants into two groups, a higher and lower functional level group. The lower functioning group, categorized by GMFCS levels IV and V completed dimensions A, B, and C on the GMFM, and the higher functioning group, categorized by GMFCS levels I, II, and III, completed dimensions C, D, and E. The use of a screen to determine which set of functionally relevant items to administer is similar to the previously validated ‘Item Set’ approach. All individuals in this study were assessed with more items than the previously determined 13 items necessary to accurately predict motor function (Additional file 2: Fig. S2). Each group in this study was administered a set of 51 items.

### Assessments

#### Gross motor function classification system (GMFCS)

The GMFCS is a system of classifying maximum level of independent motor function, developed to describe various abilities of those with CP. The GMFCS is also useful to predict potential use of assistive devices. Over the age of five, GMFCS levels do not typically change over time, allowing levels to be a useful indication of function beyond only the current level performed [20].

#### Gross motor function measure (GMFM)

Gross motor function was evaluated using items from the GMFM-88, a criterion-referenced assessment tool validated for children five months to 16 years with CP or DS whose motor skills are at or below a five-year-old without any motor disability. Higher scores on the GMFM indicate better function [10, 11]. GMFM scores were converted to GMFM-66 scores during data processing. The GMFM-66 has expanded interpretability and is a superior choice for comparing on a common scale and longitudinal analysis. The GMFM-66 can be scored as a maximum of 100% [15]. Process of gross motor function classification and item administration is represented in Additional file 2: Fig. S2.

#### Revised upper limb module (RULM)

Upper extremity function was evaluated using the RULM. It assesses shoulder, mid-level elbow, wrist, and hand function. The RULM demonstrates good reliability and validity and was originally intended as a more sensitive scale to assess the range of upper extremity function in SMA [21]. It tests items in increasing level of difficulty that relate to activities of daily living such as placing hands on a table, bringing a cup to the mouth, and opening a small snack container. There is also an entry item that serves as an upper limb functional classification scored from zero to six, where six indicates no compensation in the shoulder abduction task. If unable to raise arms to shoulder level, participants score three or below on the entry item, with a zero indicating no useful function of hands. The entry item is not calculated into the total score, though is helpful to quickly identify upper limb function. A higher total score on the RULM indicates higher function, with a maximum score of 37 [21].

#### Pediatric evaluation of disability inventory computer adaptive test (PEDI CAT)

Mobility and daily activities were two domains captured using the PEDI CAT. There are a total of 76 daily activity items that assess various activities of daily living that occur in the home including eating, dressing, and grooming, while 105 mobility items assess movement between environments in both the home and community setting. Although the pool of items is large, no individual answers every single item. The most precise measurements occur when performance is in middle range [22]. The functional skills of the PEDI-CAT can be measured in children from birth to age 21 years and are valid in children with medical complexity in any setting [23].

#### Six minute walk test (6MWT)

The 6MWT is an objective evaluation of functional exercise capacity that measures the maximum distance a person can walk in six minutes over a 25-meter(m) course. Greater distance walked is associated with better function. This standardized, self-paced walking test is reliable and valid across multiple populations including SMA, cardiopulmonary conditions, as well as healthy and typically developing populations [24–26]. Reliability on the 6MWT has been established in chronic pediatric conditions such as, CP, cystic fibrosis, Duchenne muscular dystrophy (DMD), and spina bifida [27]. The 6MWT has been used as a primary and secondary outcome measure in several phase 3 therapeutic clinical trials in pediatric conditions like DMD and Pompe Disease [28, 29]. The percent fatigue can be calculated based on the difference and percent change between the first and last minute walked. The percent predicted distance for sex, age, height, and weight can be calculated and provides a comparison to healthy peers [26].

#### Timed up and go (TUG)

The TUG is a functional, dynamic, balance and mobility test. Participants are instructed to stand up from a chair, walk three meters, turn around, and return to sitting. A walking aid can be used if necessary [30, 31]. In addition to use in adults, the TUG has been studied in typically developing children and those with disabilities, with high reliability found in the typical children as well as children with CP and spina bifida. The TUG has been successfully used in children as young as 3 years old and is a measure of functional mobility and is responsive to change over time [32].

#### Ten meter walk/run (10MWR)

During the 10MWR, participants are instructed to walk or run 10 meters as quickly and safely as possible. Motivation is used to encourage fast walking or running when possible [33]. Both feet begin together with toes at the start line. To account for a ramp down period and prevent deceleration impacting final time, participants are instructed to run two meters beyond the 10-meter mark. Time is stopped when the second leg crosses the finish line.

### Statistical analysis

Descriptive statistics were used to present participant characteristics. Analysis of variance (ANOVA) was used to assess differences between GMFM-66 and GMFCS levels as well as between GMFM-66 and caregiver reported ambulatory status. Tukey’s post-hoc test was used to assess group differences in the ANOVA analysis. Normality was assessed using the Shapiro-Wilk test. Independent sample t-tests were used to analyze sex differences on the GMFM-66, and differences in performance between the two most frequent genotypes assessed. Pearson correlation coefficients were used to evaluate the bivariate relationship between the GMFM-66 and all motor function tests except TUG and 10MWR where Spearman correlation coefficient was used since these variables were not normally distributed. IBM SPSS statistics version 28 was used to analyze the data, with correlations considered significant at an alpha of *p* < 0.05.

## Results

Thirty-eight individuals with pathogenic variants in *PPP2R5D* were evaluated. There were 10 genetic variants identified among the individuals evaluated, though the two most common were Glu198Lys (n = 19, 50.0%) and Glu200Lys (n = 7, 18.4%) (Table 1). The median age of participants was 8.0 years with a greater number of females (n = 23; 60.5%) than males (n = 15; 39.5%) (Table 2).

**Table 1:** Frequency of genetic variants

Genetic variant	Frequency
Glu198Lys	19
Glu200Lys	7
Glu420Lys	4
Asp251Ala	2
Asp251His	1
Asp251Try	1
Asp251Val	1
Glu197Lys	1
Glu200_Pro201delinsGlyHis	1
Trp207Arg	1
Total	38

Frequency of the various genetic variants found in the 38 individuals with *PPP2R5D* related neurodevelopmental disorder who were evaluated for motor function testing.

**Table 2:** Participant characteristics and performance on gross motor and upper limb assessments

Measure	Median (range)
Age (years)	7.97 (1–27)
Sex (% female)	60.5
*GMFCS (n, %)*	
Level I	21 (55.3%)
Level II	5 (13.2%)
Level III	6 (15.8%)
Level IV	5 (13.2%)
Level V	1 (2.6%)
GMFM-66	60.60 (21.1–96.0)
RULM	27 (8–37)
10MWR (s)	6.18 (2.75–22.19)
TUG	8.19 (5.06–33.40)
6MWT distance (m)	289 (230–481)
6MWT (% predicted)	54.85 (30.6–71.7)
PEDICAT daily activities	49 (37–63)
PEDICAT mobility	62 (36–70)

Performance for total participants is represented as median and range unless otherwise stated. Gross motor function classification system (GMFCS), Gross Motor Function Measure-66 (GMFM-66), Revised Upper Limb Module (RULM), 10-meter walk/run (10MWR), 6-minute walk test (6MWT)-distance and % predicted, Pediatric evaluation of disability inventory computer adapted version (PEDICAT)—daily activities and mobility domains scaled scores.

**Fig. 1:**
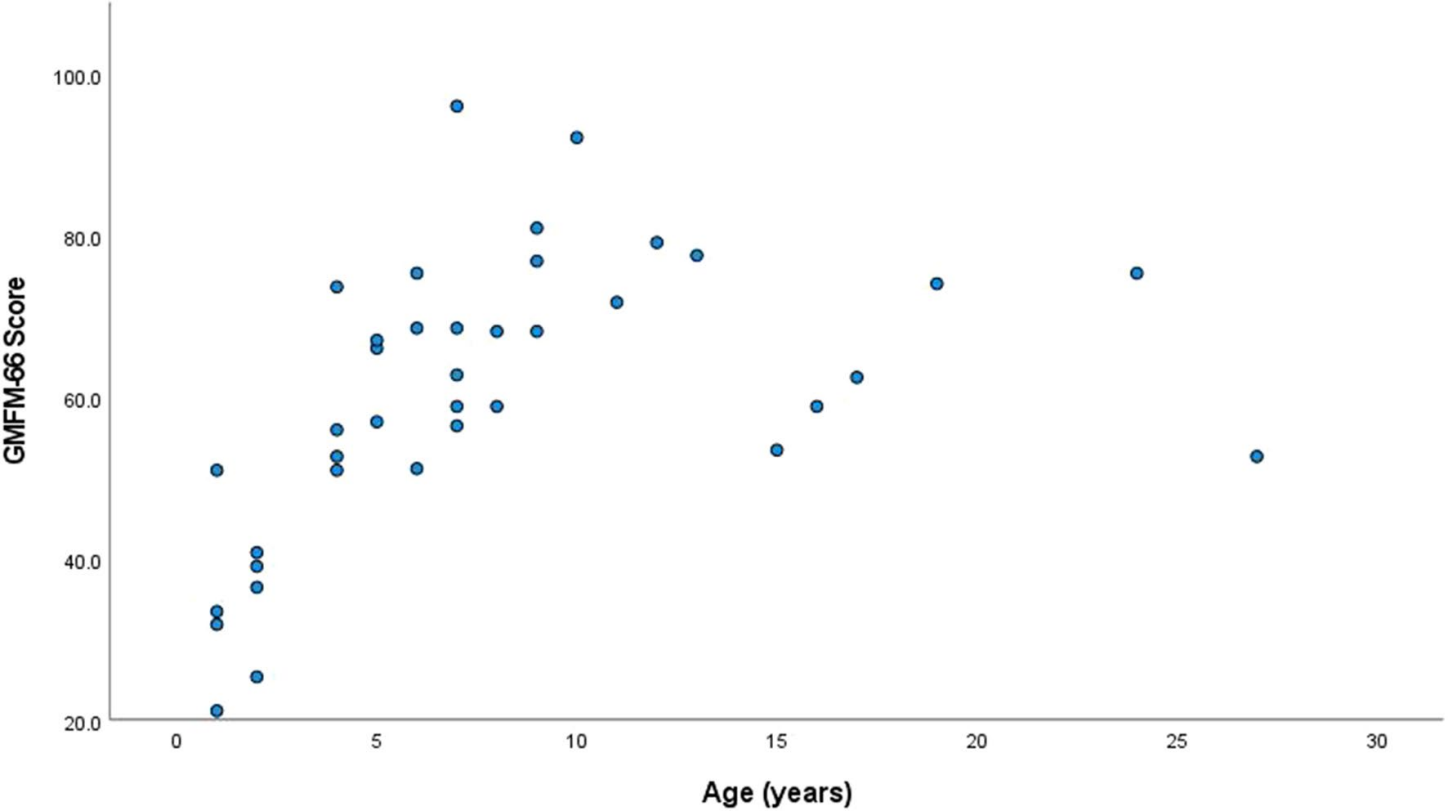
Gross Motor Function Measure-66 scores increase with age until about 10 years and then plateau

The GMFM was completed by all 38 individuals (Dimensions A–C: n = 7, Dimensions C–E: n = 31). Many of those evaluated were classified as high level in gross and upper extremity function. The majority of individuals, 21 (55.26%) were classified as GMFCS level I and were able to navigate their environment independently without an assistive device. Only one individual (2.63%) was classified as GMFCS level V. Mean score for the total group on the GMFM-66 was 60.2 (median: 60.6; range 21.1–96.0) (Table 2). GMFM-66 scores tended to increase with age until about 10 years, then gradually plateau (Figure 1). Twenty-two individuals were able to lift their arms over their head either with compensations, rated as a 5 (n = 15, 55.5%), or without compensations, rated as a 6 (n = 7, 25.9%) on the RULM entry item. A summary of total group characteristics and performance on gross and upper limb assessments can be found in Table 2.

### Concurrent validity

**Fig. 2:**
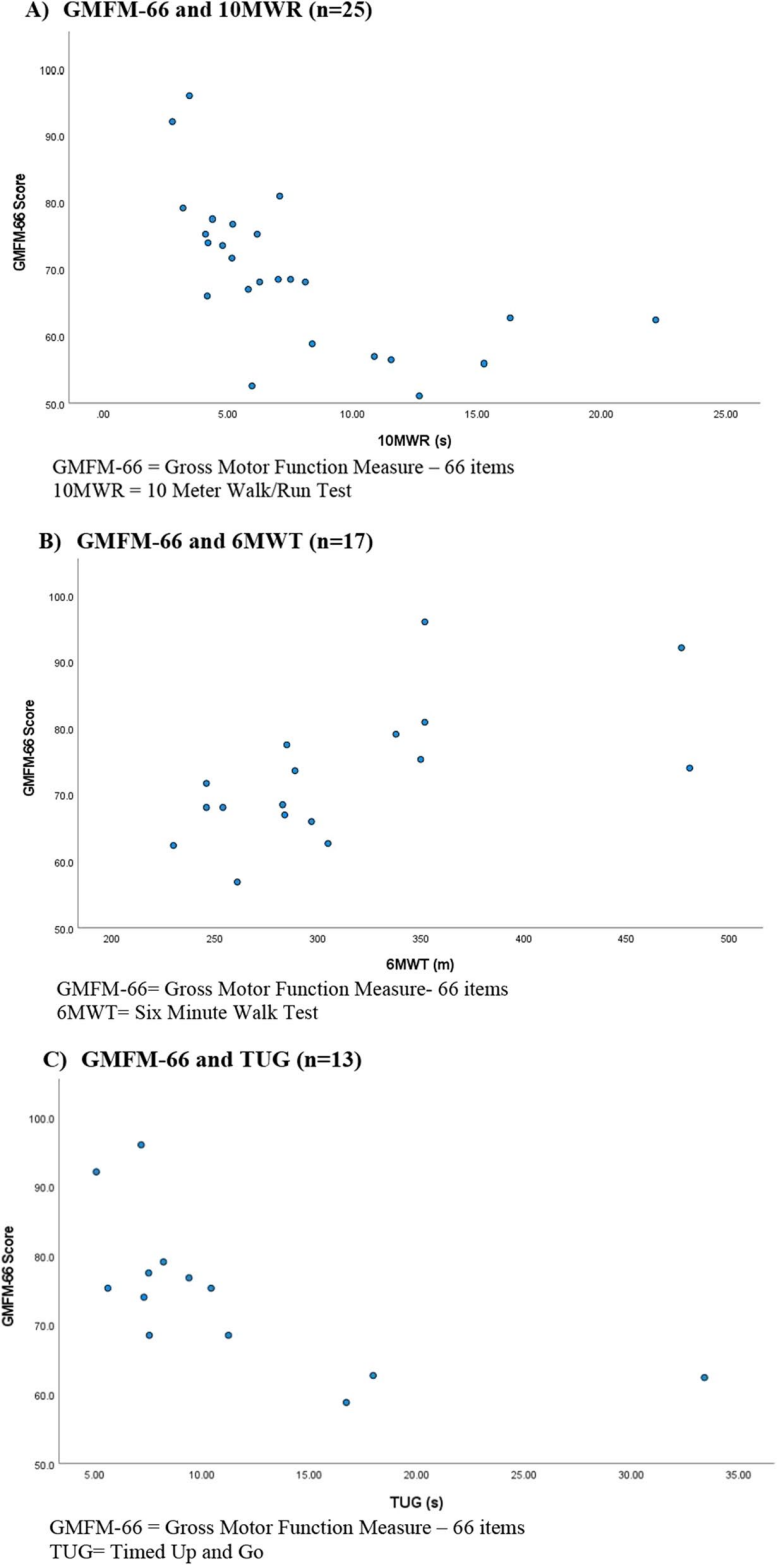
**A** The Gross Motor Function Measure-66 is inversely associated with 10 meter walk/run time. Those who score higher, walk or run faster (*p* < 0.001). **B** The Gross Motor Function Measure-66 scores are associated with the distance walked on the Six Minute Walk Test. Those who scored higher walked further (*p* = 0.006). **C** The Gross Motor Function Measure-66 scores are inversely associated with the timed up and go. Those who scored higher on the GMFM-66 were able to complete the TUG faster (*p* = 0.003)

Associations between GMFM-66 and assessments of ambulatory and upper limb function and PEDICAT are included in Table 3. There was a strong inverse association between GMFM-66 and 10MWR time (r = −0.733; *p* < 0.001) as well as TUG time (r = −0.747; *p* = 0.003) where higher scores on the GMFM-66 were correlated with faster performance on both 10MWR and TUG. A strong positive association was found between the GMFM-66 and the RULM (r = 0.763; *p* < 0.001). There was a moderate positive association between the GMFM-66 and 6MWT distance (r = 0.633; *p* = 0.006). Scatter plots comparing the GMFM-66 scores and performance on timed function tests are depicted in Fig. 2a–c. There was a strong positive association between the GMFM-66 and both the PEDICAT mobility domain (r = 0.885; *p* < 0.001) and daily activity domain (r = 0.822; *p* < 0.001).

**Table 3:**
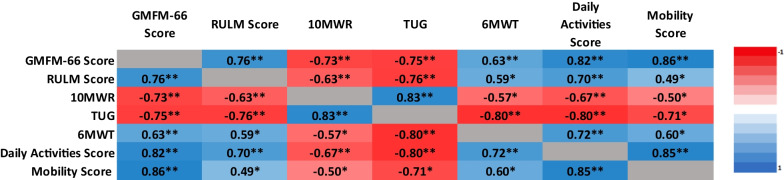
Associations between GMFM 66 and motor function tests

Heat Map showing associations between Gross Motor Function Measure-66 and related assessments of mobility. Clinician administered outcome measures including Revised Upper Limb Module, Ten Meter Walk/Run, Timed Up and Go, and Six Minute Walk Test. Caregiver reported outcome measures include two domains of the PEDICAT: daily activities and mobility. Dark warm colors represent strong associations and asterisks denote significant associations. (*p < 0.05, **p < 0.001)

### Discriminant validity

The GMFM-66 was able to discriminate individuals with *PPP2R5D* by both caregiver reported ambulatory status and GMFCS level. When GMFM-66 was compared to caregiver reported ambulatory status, a one-way ANOVA revealed there was a statistically significant difference in GMFM-66 score between at least two groups (F(2,34)=[15.454], *p* < 0.001). Those who are independent ambulators were found to be significantly different from those who used a wheelchair either part or full-time. (*p* < 0.01) There were no significant differences between those who use a wheelchair part-time and full-time (*p* > 0.05). There were no individuals who were reported by caregivers to walk with an assistive device, therefore this group was not included in analysis. When GMFM-66 was compared to GMFCS levels, a one-way ANOVA revealed there was a significant difference in GMFM-66 score between at least two groups (F(34,3639)=[25.657], *p* < 0.001). There was a significant difference between all GMFCS levels (*p* < 0.05), except levels II and III. GMFCS level V was excluded from analysis since there was only one participant in the group. GMFM-66 scores were not significantly different between males and females (p = 0.237). Individuals with Glu200Lys (n = 7) performed significantly higher on the GMFM-66 than those with the Glu198Lys (n = 19) variant (*p* = 0.029). Additional file 1: Fig. S1a–c depicts the GMFM-66 scores and differences between these various groups.

## Discussion

The GMFM is a valid assessment of motor function in children and adults with pathogenic variants in *PPP2R5D*. The GMFM was the most appropriate criterion-referenced outcome measure to include in this study for its established validity in multiple similar populations and its ability to measure individuals with a wide range of motor function. To our knowledge, this is the first attempt to characterize motor function of a large cohort of individuals with *PPP2R5D* variants using standardized, quantitative assessments. Much of the previous literature describes case studies or focuses on other medical and genetic information with gaps in motor function and performance, though developmental delay is reported as frequently observed in this condition [34].

In *PPP2R5D* related neurodevelopmental disorder, the GMFM-66 demonstrated concurrent validity with assessments of ambulatory and upper limb function, specifically the 10MWR, 6MWT, RULM and TUG. We also demonstrated concurrent validity with the mobility and daily activity domains of the PEDICAT. The PEDICAT may be more representative of activities that occur in the home setting. The strong association between clinician administered assessments and caregiver reported measures further indicates that the GMFM was an appropriate choice of assessment tool. It is reasonable to conclude that both measures will be important to include as endpoints in clinical trials [35]. The GMFM-66 score was also able to discriminate between functional levels, as determined by the GMFCS as well as those assessed through caregiver report in our *PPP2R5D* cohort.

This work has limitations. Similar to other abbreviated versions, each participant performed only a subset of domains on the GMFM based on functional level due to time constraints and to minimize burden. Due to scheduling, all participants completed motor function testing in a different order at varied times of the day. This may have resulted in impacts on endurance and fatigue on motor function. Further limitations include small sample size of the cohort with heterogeneity of genetic variants in *PPP2R5D*. Although initially useful to describe functional abilities in a new population, the GMFCS may not be the most relevant classification system as the majority of individuals did not use an assistive device. A revised or new classification system may be important to incorporate into future work to more accurately classify motor function in individuals with genetic variants in *PPP2R5D.*

The GMFM is a valid tool to evaluate and characterize motor function in children and adults with *PPP2R5D* related neurodevelopmental disorder. This study represents cross sectional data from a group of individuals ranging in age. In our cross-sectional analysis, there was improvement on the GMFM with age, up until about 10 years. For the best understanding of change over time, longitudinal data are still needed. Future work could compare the sensitivity of of this modified approach to the GMFM-88 or GMFM-66. Additional future studies should focus on longitudinal assessments in the same group of individuals annually, using the same methodology to understand the natural history. This will allow better interpretation of GMFM changes with age. Continued collection of outcome measures in a natural history cohort will also help to develop standard of care treatment. With future clinical trials, the GMFM could serve useful as a motor function endpoint in *PPP2R5D* related neurodevelopmental disorder and additionally as part of clinical management for this and other genetically determined neurodevelopmental disorders.

### Supplementary Information

**Additional file 1**. **Fig. S1**. **A** The Gross Motor Function Measure-66 scores are able to discriminate by ambulatory status as reported by caregivers. Three distinct groups emerge: independent ambulators, partial wheelchair users, and full-time wheelchair users. There is a significant difference between independent ambulation and partial wheelchair use (*p* < 0.01). No caregiver reported use of an assistive device to walk. **B** The Gross Motor Function Measure-66 scores are able to discriminate between Gross Motor Function Classification System Levels. There is a significant difference between all groups (*p* < 0.05) except between GMFCS II and III. **C** The Gross Motor Function Measure-66 scores differ between the two most frequent genetic variants in this cohort, with Glu200Lys (n = 7) performing significantly better (*p* = 0.029) than Glu198Lys (n = 19).

**Additional file 2**. Flowchart demonstrating the process of gross motor function classification and item administration. Gross motor function classification system (GMFCS) was used to split all participants into a high or low functioning group. The dimensions and items that were administered to each group are presented. A portion of those items were then used to calculate a modified Gross Motor Function Measure (GMFM)-66 score

## Data Availability

The dataset(s) supporting the conclusions of this article is(are) included within the article (and its additional file(s)).

## Abbreviations

*PPP2R5D*: Protein phosphatase two regulatory subunit B’ delta
PPP2A: Phosphatase-2A
ID: Intellectual disability
ASD: Autism spectrum disorder
ASO: Antisense oligonucleotides
SMA: spinal muscular atrophy
GMFM: Gross motor function measure
CP: Cerebral palsy
DS: Down syndrome
GMAE: Gross motor ability estimator
PEDI-CAT: Pediatric evaluation of disability inventory computer adapted version
GMFCS: Gross motor function classification system
RULM: Revised upper limb module
6MWT: Six minute walk test
TUG: Timed up and Go
10MWR: 10 meter walk/run
ANOVA: Analysis of variance
