# Supplementary material for: Validation of a modified version of the gross motor function measure in PPPR5D related neurodevelopmental disorder
Source: Orphanet J Rare Dis. 2024 Feb 7;19:45. doi: 10.1186/s13023-024-03067-3 (PMC10848481; doi:10.1186/s13023-024-03067-3)
Supplement: Supplementary file 1 — Additional file 1. Fig. S1. A The Gross Motor Function Measure-66 scores are able to discriminate by ambulatory status as reported by caregivers. Three distinct groups emerge: independent ambulators, partial wheelchair users, and full-time wheelchair users. There is a significant difference between independent ambulation and partial wheelchair use (p < 0.01). No caregiver reported use of an assistive device to walk. B The Gross Motor Function Measure-66 scores are able to discriminate between Gross Motor Function Classification System Levels. There is a significant difference between all groups (p < 0.05) except between GMFCS II and III. C The Gross Motor Function Measure-66 scores differ between the two most frequent genetic variants in this cohort, with Glu200Lys (n = 7) performing significantly better (p = 0.029) than Glu198Lys (n = 19). [file 13023_2024_3067_MOESM1_ESM.docx]

**Supplemental Figure 1**

**A) GMFM-66 scores grouped by caregiver-reported ambulatory status (n=38)**


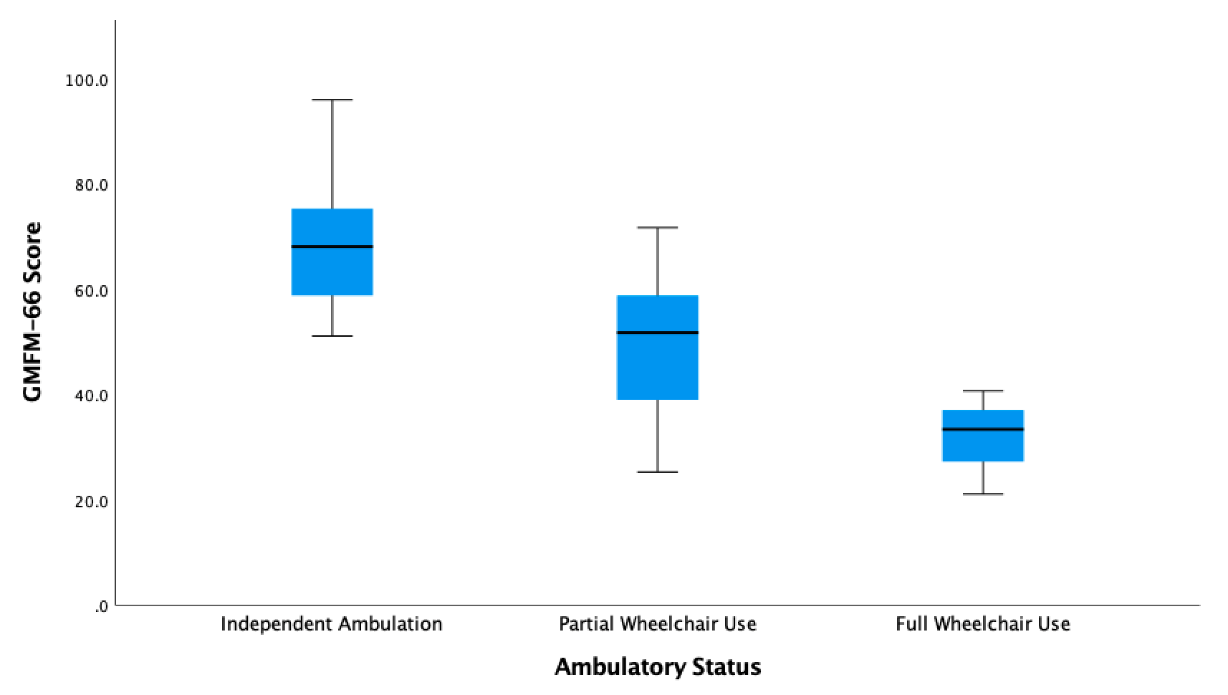


GMFM-66 = Gross Motor Function Measure – 66 items


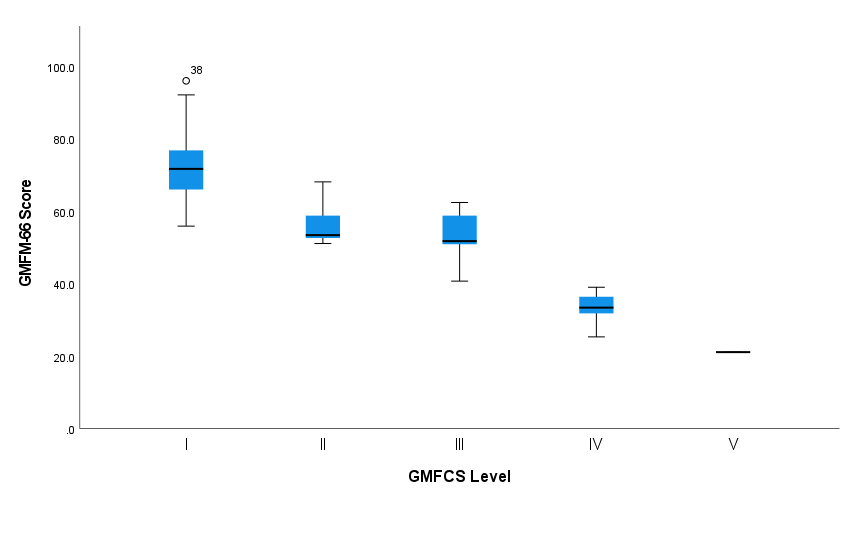
**B) GMFM 66 grouped by GMFCS level**

GMFM-66 = Gross Motor Function Measure – 66 items

GMFCS = Gross Motor Functional Classification System

**C) GMFM-66 scores grouped by the two most common *PPP22R5D* variants included in this cohort (Glu198Lys and Glu200Lys)**


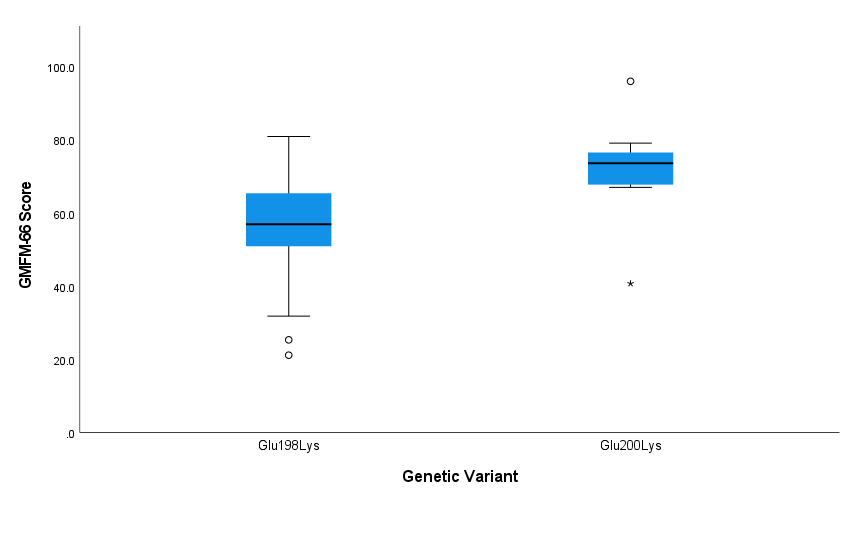


GMFM-66 = Gross Motor Function Measure – 66 items
