# Supplementary figures and images for: Validation of a modified version of the gross motor function measure in PPPR5D related neurodevelopmental disorder
Source: Orphanet J Rare Dis. 2024 Feb 7;19:45. doi: 10.1186/s13023-024-03067-3 (PMC10848481; doi:10.1186/s13023-024-03067-3)

**Supplemental Figure 2. Methodology of Gross Motor Function item administration**


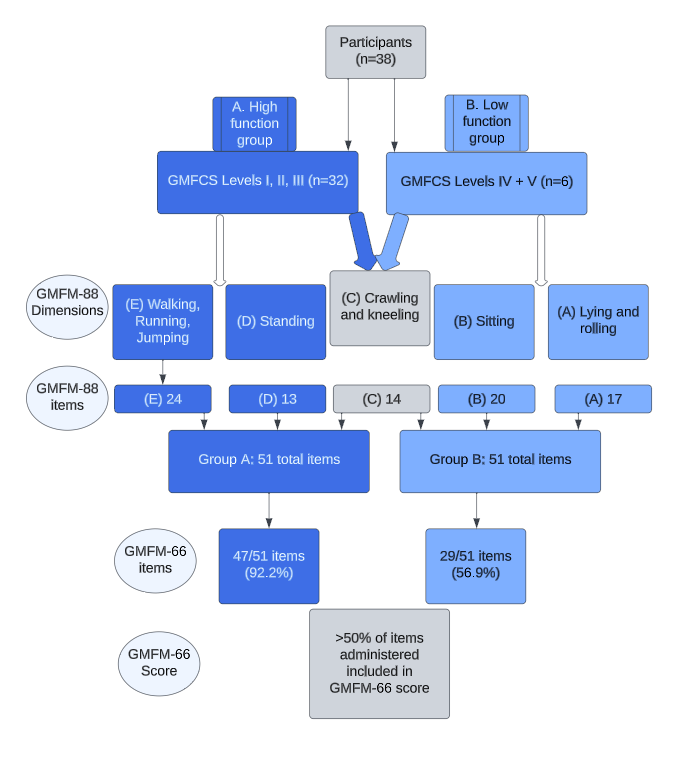

Supplement: Supplementary file 2 — Additional file 2. Flowchart demonstrating the process of gross motor function classification and item administration. Gross motor function classification system (GMFCS) was used to split all participants into a high or low functioning group. The dimensions and items that were administered to each group are presented. A portion of those items were then used to calculate a modified Gross Motor Function Measure (GMFM)-66 score [file 13023_2024_3067_MOESM2_ESM.docx]
